# White Matter Abnormalities Associated With Prolonged Recovery in Adolescents Following Concussion

**DOI:** 10.3389/fneur.2021.681467

**Published:** 2021-06-24

**Authors:** João Paulo Lima Santos, Anthony P. Kontos, Sarrah Mailliard, Shawn R. Eagle, Cynthia L. Holland, Stephen J. Suss, Halimah Abdul-waalee, Richelle S. Stiffler, Hannah B. Bitzer, Nicholas A. Blaney, Adam T. Colorito, Christopher G. Santucci, Allison Brown, Tae Kim, Satish Iyengar, Alexander Skeba, Rasim S. Diler, Cecile D. Ladouceur, Mary L. Phillips, David Brent, Michael W. Collins, Amelia Versace

**Affiliations:** ^1^Department of Psychiatry, Western Psychiatric Hospital, University of Pittsburgh, Pittsburgh, PA, United States; ^2^Department of Orthopaedic Surgery, University of Pittsburgh Medical Center (UPMC) Sports Concussion Program-University of Pittsburgh, Pittsburgh, PA, United States; ^3^Department of Radiology, Magnetic Resonance Research Center, University of Pittsburgh, Pittsburgh, PA, United States

**Keywords:** adolescence, concussion, predictors, recovery, diffusion MRI

## Abstract

**Background:** Concussion symptoms in adolescents typically resolve within 4 weeks. However, 20 – 30% of adolescents experience a prolonged recovery. Abnormalities in tracts implicated in visuospatial attention and emotional regulation (i.e., inferior longitudinal fasciculus, ILF; inferior fronto-occipital fasciculus, IFOF; uncinate fasciculus; UF) have been consistently reported in concussion; yet, to date, there are no objective markers of prolonged recovery in adolescents. Here, we evaluated the utility of diffusion MRI in outcome prediction. Forty-two adolescents (12.1 – 17.9 years; female: 44.0%) underwent a diffusion Magnetic Resonance Imaging (dMRI) protocol within the first 10 days of concussion. Based on days of injury until medical clearance, adolescents were then categorized into SHORT (<28 days; *N* = 21) or LONG (>28 days; *N* = 21) recovery time. Fractional anisotropy (FA) in the ILF, IFOF, UF, and/or concussion symptoms were used as predictors of recovery time (SHORT, LONG). Forty-two age- and sex-matched healthy controls served as reference. Higher FA in the ILF (*left*: adjusted odds ratio; AOR = 0.36, 95% CI = 0.15 – 0.91, *P* = 0.030; *right*: AOR = 0.28, 95% CI = 0.10 – 0.83, *P* = 0.021), IFOF (*left:* AOR = 0.21, 95% CI = 0.07 – 0.66, *P* = 0.008; *right*: AOR = 0.30, 95% CI = 0.11 – 0.83, *P* = 0.020), and UF (*left*: AOR = 0.26, 95% CI = 0.09 – 0.74, *P* = 0.011; *right:* AOR = 0.28, 95% CI = 0.10 – 0.73, *P* = 0.010) was associated with SHORT recovery. In additional analyses, while adolescents with SHORT recovery did not differ from HC, those with LONG recovery showed lower FA in the ILF and IFOF (*P* < 0.014). Notably, inclusion of dMRI findings increased the sensitivity and specificity (AUC = 0.93) of a prediction model including clinical variables only (AUC = 0.75). Our findings indicate that higher FA in long associative tracts (especially ILF) might inform a more objective and accurate prognosis for recovery time in adolescents following concussion.

## Introduction

Sport-related concussion represents a major public health problem in children and adolescents (1, 2). Typically, concussion symptoms resolve within the first 4 weeks of injury without any prolonged effects in this population (3). In some cases, however, symptoms persist beyond this time frame and affect functioning and academic performance (4–10). Studies investigating risk factors for persistence of concussion symptoms have identified that reporting higher severity of concussion symptoms (11–14), neurocognitive impairment (15, 16), being female (17), having a delay in the first clinical visit (11, 18), and/or history of previous concussions (17) are associated with prolonged recovery after injury. Yet, these factors cannot help quantifying the extent of the injury and if this is associated with different outcomes.

Neuroimaging can detect neural anatomical abnormalities following concussion. In recent years, studies have shown structural and functional abnormalities in the brain following concussion (19). Diffusion Magnetic Resonance Imaging (dMRI), in particular, has been used to show micro- and macro-structural abnormalities in white matter tracts (20, 21). Most dMRI studies focused on adult populations and showed that adults who sustained a concussion have lower fractional anisotropy (FA, an index of microstructural directionality of the water, reflecting the collinearity and/or integrity of the fibers in the brain) (22) in key tracts of visuospatial attention (23–26), including the inferior longitudinal fasciculus (ILF) (23), inferior fronto-occipital fasciculus (IFOF) (23, 24), and uncinate fasciculus (UF) (25, 26) in comparison to healthy controls. In addition, research showed that FA positively correlates with better cognitive performance, as measured by the Standard Assessment of Concussion, in collegiate football players (mean age [SD] = 19.2 [1.0]) in the acute stages of concussion (27). Research also demonstrated that other dMRI measurements are affected by concussion. For example, after accounting for pre-injury abnormalities in thirteen collegiate athletes, Cubon et al. showed that concussion is associated with increases in radial diffusivity (RD, a metric that reflects the displacement of water perpendicular to the principal direction) (22) in the IFOF and UF tracts, reflecting local edema, disorganization of the fiber architecture and/or abnormal white matter integrity (28). In children and adolescents, however, concussion has been associated with both increases and decreases in FA. For example, even if in pediatric populations most studies have shown higher FA and lower RD in initial stages of concussion (29–33), lower FA in the UF has been associated with poor outcomes, including prolonged recovery time and persistence of symptoms months after injury (26, 34). In one of these studies (34), King et al. showed that children reporting a persistent symptomatology 1 month after injury showed lower FA in the UF when compared to healthy controls (34). Altogether, these findings suggest that lower FA in white matter tracts implicated in visuospatial attention and emotional regulation may be associated with a persistent symptomatology. Recent dMRI methods allow the detection of collinearity and/or integrity along the entire tract (tractometry/tract-profile) and identify both focal and widespread abnormalities (35, 36), likely increasing the sensitivity of these techniques.

The aim of the present study was to expand on the extant literature and test if dMRI close to the injury can help predict recovery time up to 13 months after concussion in 12–17-year-olds. To this end, dMRI data were collected within the first 10 days of concussion. Based on days of injury until medical clearance, concussed adolescents were categorized into those with short (<4 weeks) or long (>4 weeks) recovery time. dMRI data were also collected in a sample of age- and sex-match healthy controls to aid interpretation of dMRI findings distinguishing adolescents with short from those with long recovery time. To the best of our knowledge, this is the first study to evaluate recovery time after concussion in a pediatric population followed up for 13 months after injury using a combination of tractography and tract-profile approaches. Based on the extant literature, our main hypothesis was that, in adolescents who sustained a recent concussion (up to 10 days), lower fiber collinearity in major white matter tracts previously reported to be abnormal following concussion, namely ILF, IFOF, and UF, would be associated with prolonged recovery (symptoms persisting after 4 weeks of injury). We further hypothesized that the inclusion of dMRI findings would increase the predictive value of clinical prediction models (secondary hypothesis).

## Materials and Methods

### Participants

The study was approved under an expedited protocol by the University of Pittsburgh Institutional Review Board. An initial sample of fifty adolescents (12.1 – 17.9 years; 19 [44.0%] female) with a recently (range between concussion and study entry = 1–10, mean [SD]: 7.2 [2.4] days) diagnosed concussion were recruited through the longitudinal Investigating Concussion in Adolescents at Risk for Emotion dysregulation (iCARE) study (R01MH11488101; PIs: Versace, Kontos). Eight (16%) adolescents were excluded from the analysis due to incomplete neuroimaging acquisition or loss of follow-up (for more information, see Supplementary Table 1), leaving a total of 42 (84%) concussed participants (age range = 12.1–17.9 years old; 42.9% female). The vast majority of the 42 participants showed sport-related concussion (*N* = 35, 83.3%). The remaining participants reported fall/accident (*N* = 6, 14.3%) and direct blow to the head (*N* = 1, 2.4%) as the cause of concussion. Forty-two age- and sex- matched adolescent healthy controls (HC) with no Axis-I diagnoses were recruited through a local recruitment website (https://pittplusme.org) and an ongoing neuroimaging study (i.e., Mood and Brain Circuitry in Adolescence [MBA] study [R01 MH111600, PIs: Ladouceur, Diler]). Demographic characteristics of the sample are detailed in Table 1. Exclusion criteria are described in the Supplementary Methods.

**Table 1:** Demographic and clinical characteristics.

**Demographic characteristics^a^**	**Total sample (*N* = 42)**	**Short (*N* = 21)**	**Long (*N* = 21)**	***t*_**(40)**_ or χ2**	***P-value*^b^**
Age, mean [SD], y	15.5 [1.7]	15.6 [1.7]	15.5 [1.8]	0.1	0.947
**Sex**					
Male, No. (%)	24 (57.1%)	15 (71.4%)	9 (42.9%)	3.5	*0.061*
Female, No. (%)	18 (42.9%)	6 (28.6%)	12 (57.1%)		
**Race**					
Caucasian, No. (%)	37 (88.1%)	18 (85.7%)	19 (90.5%)	0.2	0.634
Non-Caucasian, No. (%)	5 (11.9%)	3 (14.3%)	2 (9.5%)		
**Clinical characteristics**	**Total sample (*****N*** **=** **42)**	**Short (*****N*** **=** **21)**	**Long (*****N*** **=** **21)**	***t***_**(40)**_	*****P-value***^b^**
**Composite scores**					
Verbal memory, mean [SD]	75.9 [16.2]	81.0 [14.5]	70.9 [16.5]	2.1	**0.042**
Visual memory, mean [SD]	68.2 [17.2]	74.8 [17.4]	61.7 [14.7]	2.6	**0.012**
Visual motor speed, mean [SD]	32.8 [8.7]	34.7 [8.3]	30.9 [8.8]	1.4	0.164
Reaction time, mean [SD]	0.7 [0.2]	0.7 [0.2]	0.7 [0.2]	0	0.993
**Symptom factors**					
Affective factor, mean [SD]	1.2 [1.5]	0.8 [1.2]	1.7 [1.6]	−2.2	**0.037**
Somatic factor, mean [SD]	1.7 [2.1]	1.2 [1.7]	2.2 [2.2]	−1.6	0.116
Sleep factor, mean [SD]	1.0 [1.7]	0.5 [1.0]	1.6 [2.1]	−2.1	**0.046**
Cognitive-migraine-fatigue factor, mean [SD]	8.0 [4.1]	6.2 [3.5]	9.8 [3.9]	−3.1	**0.003**
VOMS total symptom score, mean [SD]	51.8 [39.0]	36.2 [23.9]	67.3 [45.2]	−2.8	**0.008**
Recovery time after concussion, mean [SD]	43.3 [38.1]	15.9 [4.1]	70.7 [37.3]	−6.7	** <0.001**
Time between injury and dMRI acquisition, mean [SD]	7.0 [2.5]	7.0 [2.7]	7.1 [2.4]	−0.2	0.859
**History of previous concussions^c^**, No. (%)****					
Yes, No (%)	13 (33.3%)	8 (44.4%)	5 (23.8%)	1.9	0.173
No, No (%)	26 (66.6%)	10 (55.6%)	16 (76.2%)		
**History of headaches^d^**, No. (%)****					
Yes, No (%)	19 (47.5%)	9 (47.4%)	10 (47.6%)	<0.1	0.987
No, No (%)	21 (52.5%)	10 (52.6%)	11 (52.4%)		
**History of nausea^d^**, No. (%)****					
Yes, No (%)	7 (17.5%)	4 (19.0%)	3 (14.3%)	0.3	0.574
No, No (%)	33 (82.5%)	15 (78.9%)	18 (85.7%)		

*HC, Healthy Controls; SHORT, Short Recovery; LONG, Long Recovery; VOMS, Vestibular/Ocular-Motor Screening*.

a *There were no demographic differences between Concussed participants and Healthy Controls. For additional information, see Supplementary Table 2*.

b *P ≤ 0.050 are reported in bold characters and P-values with a trend toward statistical significance are reported in italics*.

c *Three participants had missing data*.

d *Two participants had missing data*.

### Clinical Assessments

#### Recovery Time After Concussion

Recovery time was defined as the number of days between the most recent concussion and the medical clearance (3). Participants, who were follow-up of up to 13 months (mean [SD] = 5.4 [4.9]), received medical clearance if they met the following criteria: (1) No symptoms at rest for a minimum of 24 h, (2) No provocation of symptoms with typical physical and cognitive activities, (3) Neurocognitive functioning at typical baseline, (4) Normal vestibular and oculomotor functioning, and (5) No other related medical complaints. Based on recovery time and the fact that concussion symptoms usually resolve within 4 weeks in this population (3), two concussion groups were derived: (1) SHORT, including participants who received medical clearance within the first 4 weeks (28 days) after injury, and (2) LONG, including participants who did not receive clearance in the same period.

#### Measures of Concussion

Concussion-related symptoms and impairments were assessed at study entry using the Immediate Post-concussion Assessment and Cognitive Testing (ImPACT) (37), Post-Concussion Symptom Scales (PCSS) (37), and Vestibular/Ocular-Motor Screening (VOMS) (38). ImPACT is a computerized battery that includes six neurocognitive tests (e.g., attention, verbal recognition, visual working memory, visual processing speed, reaction time, and learning) and the PCSS for symptom inventory. VOMS is a screening tool that assess vestibular and ocular motor impairments. The interviews were conducted by research staff trained to reliably administer these assessments.

##### Post-Concussion Symptoms

The PCSS for symptom inventory rated symptoms using a 0–6 Likert scale, with 0 indicating no difficulty and 1–6 indicting mild-to-severe difficulty with the symptom. As previously done (39), the scores of seventeen symptoms were combined to derive four main symptom factors: (1) cognitive-migraine-fatigue (headache, dizziness, fatigue, drowsiness, sensitivity to light, sensitivity to noise, feeling slowed down, mentally foggy, difficulty concentrating, and difficulty remembering), (2) affective (sadness, nervousness, and feeling more emotional), (3) somatic (vomiting and numbness) and (4) sleep (sleeping more than usual and sleeping less than usual).

##### Neurocognitive Tests

Four composite scores were derived using the ImPACT neurocognitive tests: (1) verbal memory (percent correct), (2) visual memory (percent correct), (3) visual motor processing speed (numerical score), and (4) reaction time (seconds). Better performance is associated with higher values in the first three composite scores and lower reaction time.

##### Vestibular and Ocular Motor Impairment

The following domains were assessed with VOMS: smooth pursuit, horizontal and vertical saccades, convergence, horizontal vestibular ocular reflex, and visual motion sensitivity. Participants used a scale of 0 (none) to 10 (severe) to report the intensity of four symptoms after the assessment of each domain: headache, dizziness, nausea, and fogginess (maximum total for each item = 40). A total symptom score was calculated by combining the score reported for each of these four symptoms (maximum total = 270).

#### Additional Clinical Measures

History of previous concussions was collected at study entry. Information regarding the presence of headaches and nausea 6 months before injury was also collected. In addition to demographic information and concussion related symptoms, psychiatric symptoms (e.g., impulsivity, anxiety, depression) were assessed with rating scales and questionnaires administered on the day of scan (40–43).

### Neuroimaging Data

MRI data were acquired up to 10 days after concussion (mean time [SD]: 7.0 [2.6] days). MRI images were reviewed by clinical board-certified radiologist to rule out major structural abnormalities. dMRI acquisition and preprocessing steps are described in the Supplementary Methods. In brief, after eddy current, subject motion, and Echo Planar Imaging (EPI) distortions correction (44, 45), dMRI bundle-specific tractography was performed using *TractSeg* (35, 36). TractSeg is a convolutional neural network approach that directly segments white matter tracts using fiber orientation distribution function (fODF) peaks, based on Multi-Shell-Multi-Tissue Constrained Spherical Deconvolution (46, 47), as proposed in Mrtrix-3 (48). Six *a priori* major white matter tracts were selected for this study: left/right ILF, left/right IFOF, and left/right UF. The contribution of other major white matter tracts (anterior thalamic radiation, cingulum bundle, fronto-pontine tract, optic radiation, parieto-occipital pontine, superior longitudinal fasciculus I-III, arcuate fasciculus, superior thalamic radiation, corticospinal tract, thalamic-premotor, thalamic-parietal, thalamic-occipital, striato-frontal, striato-premotor, and seven main bundles of the corpus callosum) to the time to recover after concussion was also explored. FA maps were calculated using Mrtrix-3 (48) tools (*dwi2tensor* and *tensor2metric*). Axial Diffusivity (AD) and Radial Diffusivity (RD) reflect the displacement of water molecules along or perpendicular to the principal diffusion direction and can help interpret FA findings (22). Thus also these measures were similarly calculated.

For each tract, overall mean and nodal values were extracted to depict the collinearity of the fibers across (mean) and along (tractometry/tract-profile) the entire tract. Tract-profile analyses allow for the characterization of dMRI properties along each tract (5 consecutive nodes, each node covering 20% of the entire tract) and thus for the identification of focal abnormalities in tracts of interest.

### Statistical Analyses

#### Level 1 Analysis: Main Hypothesis Testing (Neural Correlates of Recovery Groups)

To test our main hypothesis, we used logistic regression models in SPSS V24.0. Using six parallel regression models, we examined the main effect of mean FA in six white matter tracts of interest (left/right ILF, left/right IFOF, and left/right UF) on recovery groups (SHORT, LONG). Given the known effect of age (49–51) and sex (52) on white matter, these variables were included as covariates. In addition, the number of days between the most recent concussion and scan was used as covariate. To account for multiple testing (six tracts of interest), False Discovery Rate (FDR, *p* < 0.05) (53) was used.

##### Tract-Profile Analyses

For those white matter tracts in which, after FDR correction, there was a main effect of FA (mean FA across the entire tract) on recovery time, nodal FA values (tract-profile; 5 consecutive nodes) were used to identify if the FA differences between adolescents with SHORT and LONG recovery were focal or widespread. In the case of two or more consecutive nodes in the same tract (>40%) surviving FDR correction, mean FA was used to reflect the focal abnormality in further analyses. Mean AD and RD were also extracted from node clusters to help further interpret main FA findings.

##### Between-Group Comparisons

Additional analyses explored the group differences between LONG (or SHORT) and our healthy control group (LONG vs. HC; SHORT vs. HC) on main FA findings identified above. Analysis of covariance (ANCOVA) evaluated differences between HC and Concussed groups (SHORT combined with LONG) using the mean FA of each white matter tract of interest (left/right ILF, IFOF, and UF).

##### Recovery Time as a Continuous Measure

As opposed to using a dichotomic approach to recovery time (SHORT, LONG), number of days to recover after concussion (continuous variable) was used in linear regression models.

#### Level 2 Analysis: Secondary Hypothesis Testing (Predictive Models)

Forward stepwise logistic regression was used to build predictive models that combined clinical, demographic, and/or neuroimaging characteristics to differentiate our main groups (SHORT, LONG). Two models were tested: (1) A model that included clinical and demographic variables significantly different between the two main groups, and (2) A second model that combined clinical, demographic, and FA abnormalities identified in *Level 1 analysis*. Receiver Operating Characteristic (ROC) curve and Area Under the Curve (AUC) were used to assess the diagnostic ability of the models (54).

##### Recovery Time as a Continuous Measure

Linear regression was used to assess the ability of the combined model to predict time to recover as a continuous variable.

#### Exploratory Analyses

In the concussion group, Pearson or Spearman correlations, as appropriate, were used to explore the relationships between mean FA of abnormalities identified in *Level 1 analysis* and composite scores, post-concussion symptom factors, and VOMS total symptom score. Two-sample Kolmogorov-Smirnov tests were used to compare the distribution of FA in these node clusters (55). In addition, analysis of variance (ANOVA) was used to evaluate the effect of previous concussions or history of headaches and nausea over the past 6 months on mean FA in each node cluster.

Given the known effect of movements on dMRI measures, the main effect of average translation and average rotation was also explored in each node cluster.

## Results

All concussed participants received medical clearance (range = 9–150 days, mean [SD] = 43.3 [38.1] days), with 50% (*N* = 21) showing recovery within 4 weeks of injury (SHORT). There were no significant differences between SHORT and LONG recovery groups regarding demographic characteristics, time between injury and scan, previous concussions, history of headaches, or nausea (Table 1). Compared to SHORT, those with LONG recovery showed lower percentage of correct answers in two out of the four neurocognitive measures (i.e., verbal and visual memory composite scores), higher severity in three out of the four post-concussion symptom factors (i.e., affective, sleep, and cognitive-migraine-fatigue symptoms), and higher VOMS total symptom score (Table 1). For additional information regarding demographic and clinical characteristics, see Supplementary Tables 2, 3.

### Level 1 Analysis: Main Hypothesis Testing (Neural Correlates of Recovery Groups)

Logistic regression models revealed that there was a significant main effect of mean FA on recovery groups (SHORT, LONG) in all white matter tracts of interest (Supplementary Table 4): (1) left ILF (adjusted odd ration; AOR = 0.36, 95% CI = 0.15–0.91, *P* = 0.030, FDR *P* = 0.030), (2) right ILF (AOR = 0.28, 95% CI = 0.10–0.83, *P* = 0.021, FDR *P* = 0.025), (3) left IFOF (AOR = 0.21, 95% CI = 0.07–0.66, *P* = 0.008, FDR *P* = 0.022), (4) right IFOF (AOR = 0.30, 95% CI = 0.11–0.83, *P* = 0.020, FDR *P* = 0.025), (5) left UF (AOR = 0.26, 95% CI = 0.09–0.74, *P* = 0.011, FDR *P* = 0.022), and (6) right UF (AOR = 0.28, 95% CI = 0.10–0.73, *P* = 0.010, FDR *P* = 0.022).

#### Focal Abnormalities in Tracts of Interest

**Figure 1:**
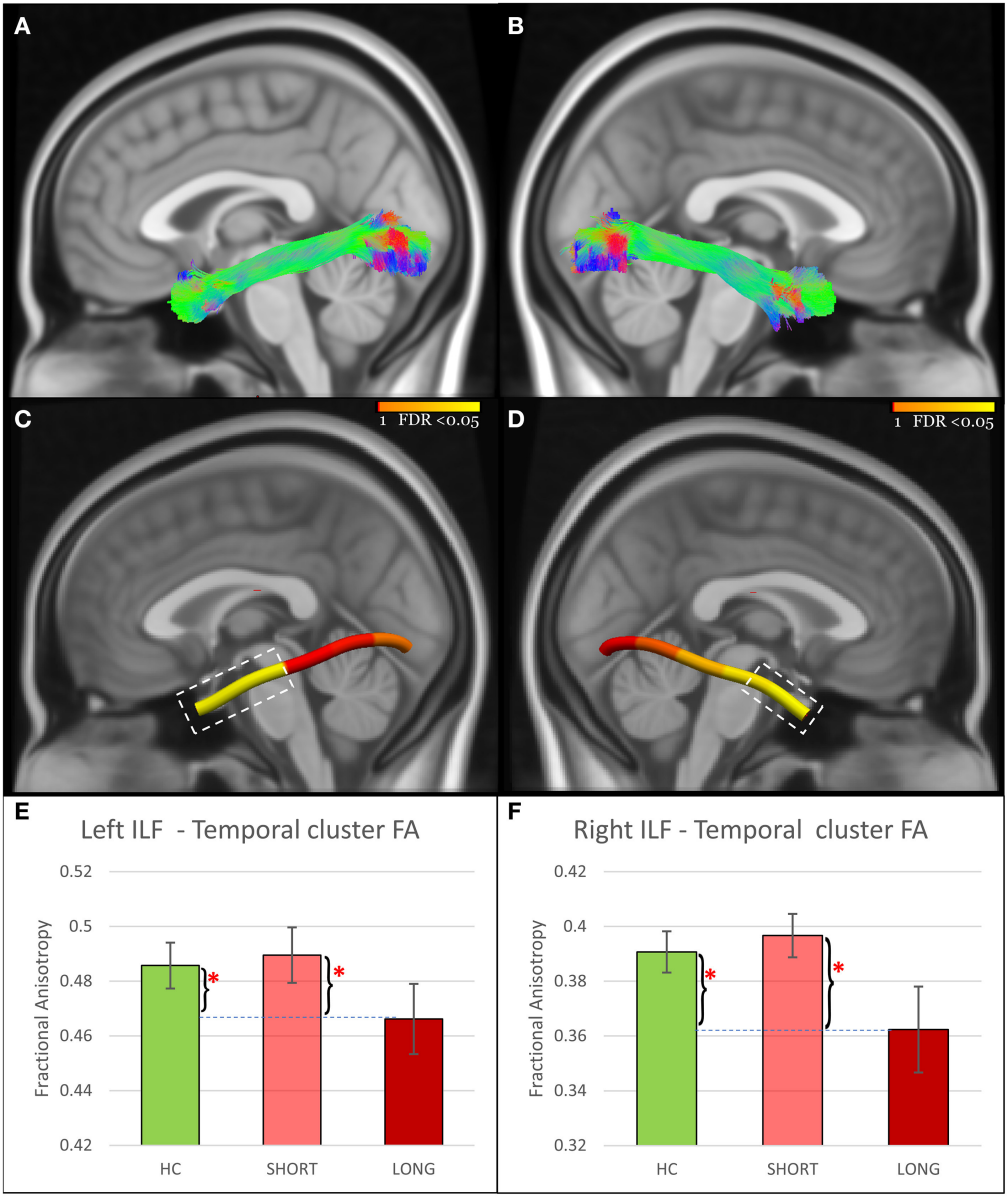
ILF findings. **(A,B)** Show the reconstructed left and right ILF using TractSeg. **(C,D)** Show node clusters in the left and right ILF. The background is the standard MNI-152 1 mm brain. Red-Yellow color bar represents the range of *p* values used in node-wise statistics after FDR correction. Error-bar plots in **(E,F)** depict the group difference upon FA in the 42 HC (green color), 21 adolescents with short recovery (SHORT, light red color), and 21 adolescents with prolonged recovery (LONG, red color) after concussion. Braces and asterisks show *p*-values that survived FDR correction. ILF, Inferior Longitudinal Fasciculus; HC, healthy control; FA, Fractional Anisotropy; FDR, False Discovery Rate.

**Figure 2:**
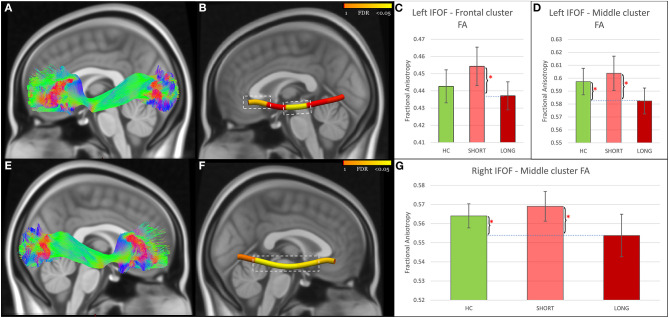
IFOF findings. **(A,E)** Show the reconstructed left and right IFOF using TractSeg. **(B,F)** Show node clusters in the left and right IFOF. The background is the standard MNI-152 1 mm brain. Red-Yellow color bar represents the range of *p* values used in node-wise statistics after FDR correction. Error-bar plots in **(C,D,G**) depict the group difference upon FA in the 42 HC (green color), 21 adolescents with short recovery (SHORT, light red color), and 21 adolescents with prolonged recovery (LONG, red color) after concussion. Braces and asterisks show *p*-values that survived FDR correction. IFOF, Inferior Fronto-Occipital Fasciculus; HC, healthy control; FA, Fractional Anisotropy; FDR, False Discovery Rate.

**Figure 3:**
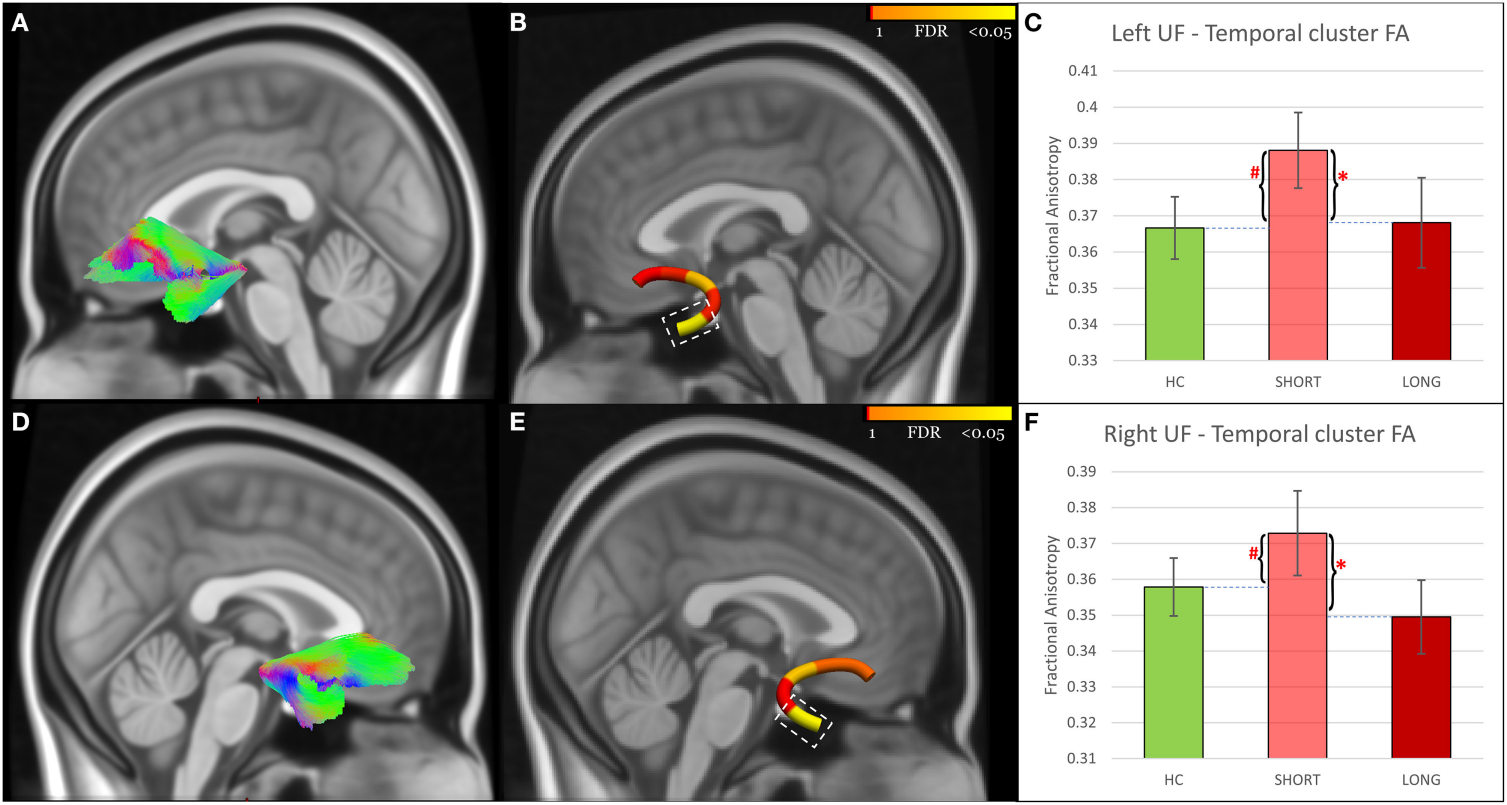
UF findings. **(A,D)** Show the reconstructed left and right UF using TractSeg. **(B,E)** show node clusters in the left and right UF. The background is the standard MNI-152 1 mm brain. Red-Yellow color bar represents the range of *p* values used in node-wise statistics after FDR correction. Error-bar plots in **(C,F)** depict the group difference upon FA in the 42 HC (green color), 21 adolescents with short recovery (SHORT, light red color), and 21 adolescents with prolonged recovery (LONG, red color) after concussion. Braces and asterisks show *p*-values that survived FDR correction. Pound signs indicate significant *p*-values that did not survive FDR correction. UF, Uncinate Fasciculus; HC, healthy control; FA, Fractional Anisotropy; FDR, False Discovery Rate.

Tract-profile analyses revealed that the FA abnormalities differentiating SHORT and LONG groups were focal (Figures 1–3): temporal portions of the left ILF (Size = 40% of the tract, AOR = 0.13, 95% CI = 0.03–0.54, *P* = 0.005, FDR *P* = 0.011), right ILF (Size = 20% of the tract, AOR = 0.16, 95% CI = 0.04–0.63, *P* = 0.009, FDR *P* = 0.011), left UF (Size = 20% of the tract, AOR = 0.25, 95% CI = 0.09–0.66, *P* = 0.005, FDR *P* = 0.011), and right UF (Size = 20% of the tract, AOR = 0.28, 95% CI = 0.11–0.70, *P* = 0.006, FDR *P* = 0.011); the middle portions of the left IFOF (Size = 20% of the tract, AOR = 0.14, 95% CI = 0.03–0.55, *P* = 0.005, FDR *P* = 0.011) and right IFOF (Size = 60% of the tract, AOR = 0.22, 95% CI = 0.07–0.67, *P* = 0.008, FDR *P* = 0.011); and the frontal portion of the left IFOF (Size = 20% of the tract, AOR = 0.30, 95% CI = 0.11–0.80, *P* = 0.016, FDR *P* = 0.016). Analyses using RD and AD measures further revealed that the lower FA in concussed participants with LONG vs. those with SHORT recovery was associated with higher RD. AD did not differ (Supplementary Table 5).

#### Between-Group Comparisons

Participants in the LONG recovery group showed lower FA in all seven node clusters in comparison to those with SHORT recovery. When compared to HC, those in the LONG recovery group showed lower FA in the temporal clusters of the left and right ILF and in the middle clusters of the left and right IFOF. There were no differences between LONG and HC in the other node clusters. However, participants in the SHORT recovery group showed higher FA than HC in the left and right UF node clusters, but these findings did not survive FDR correction. There were no differences between SHORT and HC in the other node clusters (Figures 1–3 and Supplementary Table 6).

#### Recovery Time as a Continuous Measure

Mean FA in main node clusters was negatively associated with the number of days to recover after concussion (Supplementary Table 7).

### Level 2 Analysis: Secondary Hypothesis Testing—Predictive Models

**Figure 4:**
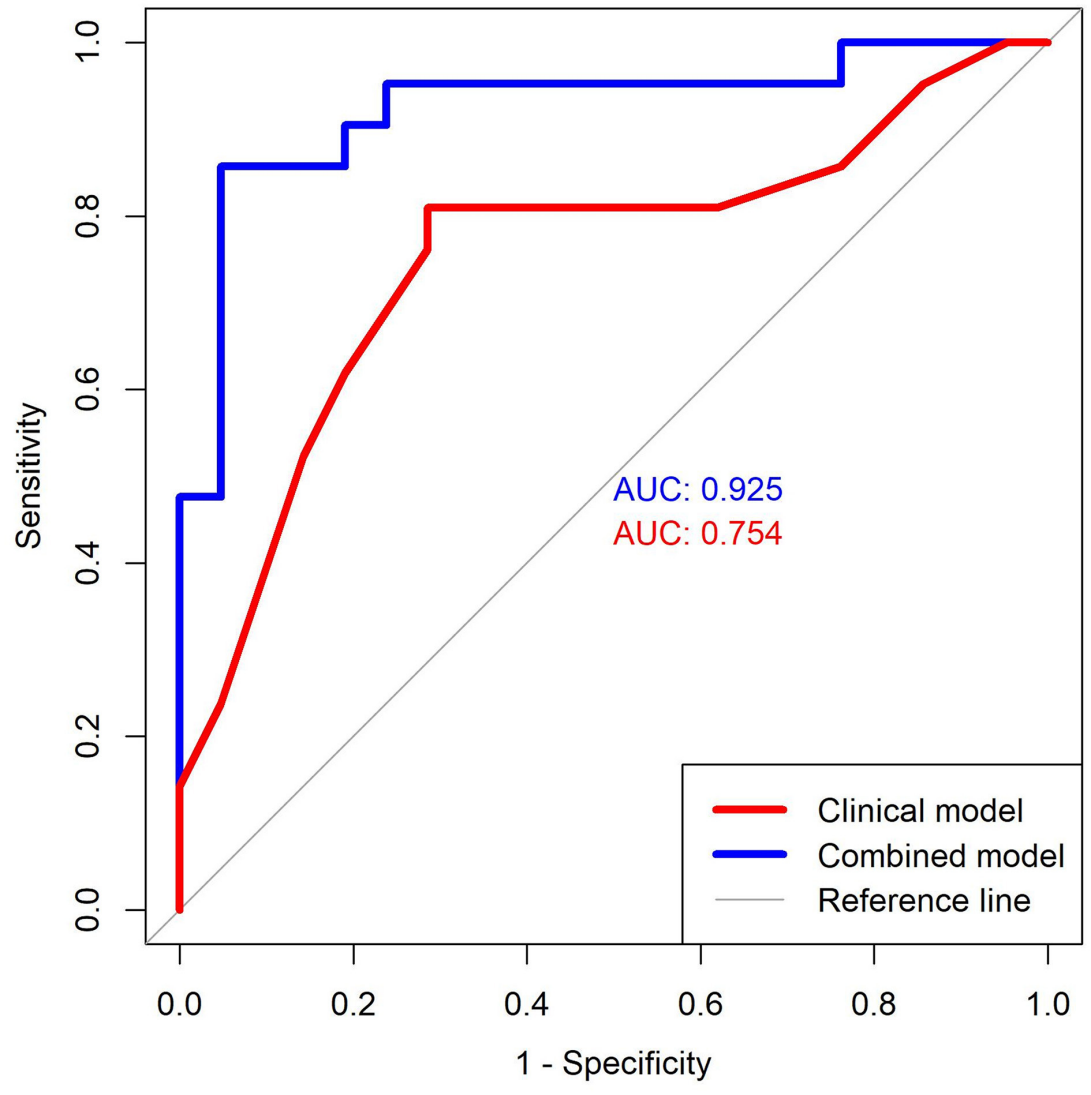
ROC curves. This figure shows the curve for the Clinical model (red color) and Combined model (blue color). The first model includes only the Cognitive-migraine-fatigue symptom factor and the second model combines the Cognitive-migraine-fatigue symptom factor with the FA of Left ILF—Temporal cluster FA and Right ILF—Temporal cluster FA. These curves were created by plotting data pairs of sensitivity/specificity and the AUC represents the discriminative ability of the test. ROC, Receiver Operating Characteristic; AUC, Area Under the Curve; ILF, Inferior Longitudinal Fasciculus.

For the model that included only clinical and demographic variables, six predictors were tested: two neurocognitive measures—verbal and visual memory composite scores, three post-concussion symptom factors—affective, sleep, and cognitive-migraine-fatigue, and the VOMS total symptom score. Only the cognitive-migraine-fatigue factor was identified. This model showed an AUC of 0.75 (Table 2 and Figure 4).

**Table 2:** Forward stepwise logistic regression results.

**Variable**	**B**	**Wald**	***P-value*^c^**	**AOR**	**95% CI**	
**Clinical model^a^**						
Cognitive-migraine-fatigue symptom factor	0.25	7.14	0.008	1.29	1.07	1.55
**Combined model^b^**						
Left ILF-Temporal cluster FA	−1.33	4.22	0.040	0.26	0.07	0.94
Right ILF-Temporal cluster FA	−1.97	4.54	0.033	0.14	0.02	0.85
Cognitive-migraine-fatigue symptom factor	0.37	6.23	0.013	1.45	1.08	1.93

*AOR, Adjusted Odds Ratio; AUC, Area Under the Curve; FA, Fractional Anisotropy; ILF, Inferior Longitudinal Fasciculus*.

a *The clinical showed an AUC of 0.754*.

b *The combined model showed an AUC of 0.925. AUC of the left and right ILF temporal clusters included in this model were 0.728 and 0.803, respectively*.

c *P ≤ 0.050 are reported in bold characters*.

For the model that combined clinical, demographic, and neuroimaging variables, thirteen predictors were tested (the same six included in the first model, in addition to the mean FA of the seven node clusters identified in *Level 1 analysis)*. Three predictors were identified: (1) Left ILF temporal cluster FA, (2) Right ILF temporal cluster FA, and (3) cognitive-migraine-fatigue factor. This second model showed an AUC of 0.93 (Table 2 and Figure 4). The difference between ROC curves was statistically significant (*P* = 0.011).

#### Recovery Time as a Continuous Measure

There was a main effect of the combined model on the number of days to recover after concussion (*F* = 6.7, *P* = 0.001).

### Exploratory Analyses

Across all concussed participants, there was no relationship between neurocognitive measures, post-concussion symptom factors, VOMS total score, history of previous concussions, headaches, or nausea with mean FA of the main findings identified in *Level 1 analysis* (for additional information, see Supplementary Table 9). In addition, recovery groups showed no differences in the distribution of FA in most of the clusters (for additional information, see Supplementary Table 9).

There was no significant effect of dMRI movements on our main findings (for additional information, see Supplementary Table 10).

Analyses involving other tracts did not yield significant findings, after accounting for multiple comparisons (for additional information, see Supplementary Table 11).

## Discussion

Findings from this study show that abnormal indices of tract microstructure—reflecting white matter integrity/organization of the fiber architecture—(i.e., lower FA and higher RD) in the ILF, IFOF, and UF are associated with higher probability of prolonged recovery (more than 4 weeks after concussion). Further analyses revealed that lower FA in the temporal ILF and middle IFOF distinguished concussed participants with a prolonged recovery from healthy participants. Notably, the inclusion of the dMRI findings in a clinical prediction model significantly increased the overall sensitivity and specificity.

In support of our main hypothesis, findings indicate that focal changes in the microstructural architecture of ILF, IFOF, and UF can help differentiate adolescents with prolonged recovery from those with short recovery (and healthy controls). The ILF is a long associative tract that connects occipital to anterior temporal regions of the brain and has been associated mainly with visuospatial attention, as well as high-order cognitive functions such as reading fluency and comprehension (56). Studies have shown that this tract develops earlier and faster than other associative tracts, with a peak around 11 years (50, 56–58). Thus, it is possible that when concussion-related lesions occur after this age (as in our sample; mean age [SD] = 15.5 [1.7] years), adolescents might need more time to recover from concussion, especially for those brain functions associated with this tract. Intervention strategies aiming to strengthen attentional processes might be able to promote/reverse plasticity of the ILF, as shown by lesion studies (59–61). The IFOF is also a long associative tract that, bypassing the temporal lobe, connects occipital to frontal cortical regions. Similar to ILF, IFOF has been implicated in visual processing, spatial attention, and higher cognitive functions, such as reading fluency and comprehension (62–64). The UF connects frontal and temporal regions of the brain and plays an important role in emotional regulation (65) (e.g., monitoring emotional distractors and redirecting attention in the context of goal-directed behavior). Within this network, the IFOF can be considered a direct path that, by promptly feeding visual information to the prefrontal cortex, carries context-relevant information for higher cognitive functioning (e.g., decision making). The ILF and UF can be considered the indirect path by which the anterior temporal pole upgrades these visual stimuli with salience (salience attribution) to then feed-forward this “enriched” information to the prefrontal cortex for additional context. Feed-back inputs (from the prefrontal cortical to temporo-occipital regions) is also mediated by these tracts. Supplementary analyses also showed that other white matter tracts involved with visual processing, visuospatial attention and executive functions (e.g., superior longitudinal fasciculus, optic radiation) (66–68) were associated with prolonged recovery, but did not survive multiple comparison correction. Studies have shown that white matter abnormalities are associated with different outcomes in concussion (26, 34, 69); however, to the best of our knowledge, this is the first study to use tractography to identify tract-specific abnormalities that are associated with prolonged recovery in adolescents and show that concussion might not affect white matter tracts uniformly. Understanding the properties along the tract is particularly important in children and adolescents, as brain maturation also does not happen uniformly (70). For example, a recent study in collegiate athletes (18.9 [0.9] years) using tract-based spatial statistical (TBSS) showed that having higher RD in the 24–48 h after concussion was associated with prolonged recovery (up to 27 days) after concussion. However, no tract-profile approach was used to inform on possible pathophysiologic mechanisms following concussion (71). Our findings indicate that abnormalities near the gray-white matter boundary in frontal and temporal regions are particularly important for recovery after concussion. This interface appears to be especially vulnerable to injury due to the different physical properties of gray and white matter and distinct responses to forces secondary to the impact (72, 73). In addition, previous studies have reported that perfusion abnormalities (e.g., reduced blood flow and volume) in frontal and temporal lobes after concussion are associated with worse outcomes (74). Interestingly, our findings in the medial portion of the IFOF sits in proximity of the temporal lobe, suggesting that—depending on the magnitude of the injury—altered perfusion in this region might also affect tracts that pass by this region to connect other lobes.

In support of our secondary hypothesis, we found that indices of white matter abnormalities did improve the performance of our clinical prediction model. Symptoms clustering in the cognitive-migraine-fatigue factor are common following concussion and have been associated with prolonged recovery in pediatric population (39, 75, 76). The ILF, as previously mentioned, is associated with visuospatial attention (56). In this study, each predictor (i.e., cognitive-migraine-fatigue factor, left ILF temporal cluster, and right ILF temporal cluster) individually showed high sensitivity and specificity to differentiate adolescents with short from those with prolonged recovery; however, the inclusion of the left/right ILF increased the predictive value of the clinical model, suggesting a potential role for dMRI in the prediction of different clinical outcomes in concussion.

There were limitations in this study. (1) Although we employed a combination of tractography and tract-profile approaches to assess white matter correlates of prolonged recovery following concussion in adolescents, as noted above, this study did not include pre-injury dMRI data. To address this limitation, we included a healthy control group as normative reference, but this approach cannot rule out the possibility that these white matter abnormalities existed before concussion. (2) Although this study yielded compelling evidence of neural correlates of prolonged recovery following concussion, future studies with larger samples are needed to replicate these findings and further show the extent to which dMRI can help predict recovery time in clinical practice. (3) Future studies should include a neuroimaging longitudinal design with repeated dMRI scans to evaluate the effect of concussion on developmental trajectories of different white matter tracts. (4) This study included adolescents in the acute/subacute stages of concussion. As such, the white matter characteristics identified during this period might not apply when including asymptomatic participants in later stages of recovery. Continued follow-up of the present sample and the inclusion of a follow-up scan will allow us to better understand the relationship between white matter and concussion in acute/subacute and chronic stages of concussion.

To our knowledge, this is the first study to identify focal abnormalities in white matter tracts that can help to distinguish between short and prolonged recovery. Our findings indicate that the integrity of white matter tracts involved in visuospatial attention is particularly important for a prompt recovery after injury. Identification of these neural correlates deepens our understanding of the pathophysiological mechanisms associated with concussion and the potential role of dMRI as an objective measure in concussion recovery. In addition, our findings suggest that the combination of clinical and dMRI measures might improve prediction and be useful for clinical decision-making and early intervention strategies.

## Data Availability Statement

The raw data supporting the conclusions of this article will be made available by the authors, without undue reservation.

## Ethics Statement

The studies involving human participants were reviewed and approved by the Institutional Review Board at the University of Pittsburgh. Written informed consent to participate in this study was provided by the participants' legal guardian/next of kin.

## Author Contributions

JL, AK, SM, CH, SS, HA-w, RS, HB, NB, AC, CS, and AV contributed to the conception, design, acquisition, and analysis of the data. JL, AK, and AV conceptualized and designed the article. JL, AK, SM, SE, CH, SS, HA-w, RS, HB, NB, AC, CS, AB, TK, SI, AS, RD, CL, MP, DB, MC, and AV contributed to manuscript revision and approved the submitted version. All authors contributed to the article and approved the submitted version.

## Conflict of Interest

MP declares a one-time honorarium from Sunovion in November 2016. DB receives research support from NIMH, AFSP, the Once Upon a Time Foundation, and the Beckwith Foundation, receives royalties from Guilford Press, from the electronic self-rated version of the C-SSRS from eRT, Inc., and from performing duties as an UptoDate Psychiatry Section Editor, receives consulting fees from Healthwise, and receives Honoraria from the Klingen-stein Third Generation Foundation for scientific board membership and grant review. MC is a former Co-owner and Board Member of ImPACT Applications (relationship ended 12/16/19). The remaining authors declare that the research was conducted in the absence of any commercial or financial relationships that could be construed as a potential conflict of interest.
