# Supplementary material for: White Matter Abnormalities Associated With Prolonged Recovery in Adolescents Following Concussion
Source: Front Neurol. 2021 Jun 24;12:681467. doi: 10.3389/fneur.2021.681467 (PMC8264142; doi:10.3389/fneur.2021.681467)
Supplement: Supplementary file 1 [file Data_Sheet_1.docx]

**SUPPLEMENTAL MATERIALS**

**Participants**

Eight adolescents were excluded from the analysis due to problems during the neuroimaging acquisition (two) or loss to follow-up (six). There were no differences regarding age, sex, and race between included and excluded concussed participants. Demographic characteristics are detailed in Supplemental Table 1.

iCARE and MBA studies share overlapping inclusion/exclusion criteria for the recruitment of healthy participants and use the same dMRI sequence and scanner. Exclusion criteria were: loss of consciousness over 5 minutes; neurological, neurodevelopmental or systemic medical (e.g., metabolic, chronic inflammatory) disease; personal history of major psychiatric disorders; current alcohol and illicit substance abuse/dependence (past three months); left/mixed handedness; IQ below 70; contraindication to participating in MRI, and intoxication or use of illicit substances (except cannabis) in urine tests on the day of the scan. History of major psychiatric disorders was excluded using the Mini International Neuropsychiatric Interview for children and adolescents (MINI-KID).[1,2]

**Clinical assessments**

Additional clinical measures

At the time of the scan, 4 dimensions of psychiatric symptoms were collected for all participants: 1. Barratt Impulsiveness Scale (BIS),[3] 2. Screen for Child Anxiety Related Disorders (SCARED),[4] 3. Children's Affective Lability Scale (CALS),[5] and 4. Children's Depression Rating Scale (CDRS).[6]

**Neuroimaging**

MRI Acquisition.

Images were acquired on a 3T Siemens Prisma at the Magnetic Resonance Research Center, University of Pittsburgh Medical Center Health System, USA. A standard body coil was used for RF transmission, while MRI signal was acquired with a Siemens 32RF channels receiver. Anatomical images covering the entire brain were acquired using an axial 3D MPRAGE sequence (Echo Time(TE)/Repetition time (TR)=3.17ms/1520ms; flip angle=8,1; 176 1mm-thick slices; matrix size=256x176; time: 4’5”). A single-shot spin-echo planar imaging (SE-EPI) sequence was acquired with 200 optimized non-colinear diffusion-weighting gradient directions (34volumes with b=750s/mm2, 69 volumes with b= 1000 s/mm2, and 106 volumes with b= 2500 s/mm2) and 12 reference volumes with b=0 s/mm2 (repetition time (TE/TR)=90ms/3000ms, flip angle=90, field-of-view (FOV)=256x256, sixty-four 2mm thick slices, no gaps, multiband factor=4, matrix size= 128x12, acquisition time=13’5"). In accordance with a forward-reverse protocol, the acquisition was collected twice with opposite phase encoding directions (P>>A and A>>P). The full brain MRI protocol can be found in the study website (https://www.versacelabs.com/icare).

Preprocessing.

Diffusion-weighted images were corrected for eddy current, subject motion, and EPI distortion using topup and eddy,[7,8] within FMRIB's Software Library (FSL). Six movement parameters, including average volume-by-volume translation and rotation in the x, y and z plane, were computed as previously proposed[9] in each participant. Absolute values were first derived for each parameter. Then, averaged translation and averaged rotation were extracted for each participant to examine if there was a main effect of movements on main findings.

## SUPPLEMENTAL RESULTS

**Demographic and clinical characteristics.**

Concussed and healthy controls groups showed no demographic differences (Supplemental Table 2). However, concussed participants showed higher BIS total score. In addition, there was no difference in psychiatric symptoms between SHORT and LONG groups (Supplemental Table 3).

**Exploratory Analyses.**

In the concussion group, the FA of the left ILF temporal cluster was positively correlated with SCARED total score. In addition, the FA of the right UF temporal cluster was positively correlated with verbal and visual memory composite scores and negatively correlated with VOMS total score. However, none of these correlations survived FDR correction (P > 0.050).

Additional analyses revealed that the FA of the Left IFOF Middle cluster was negatively correlated with average rotation, but this finding did not survive FDR correction (P > 0.050). Analyses also revealed that the mean FA of the right Arcuate fascicle, Corpus Callosum bundles (anterior midbody, posterior midbody, and splenium), right Optic radiation, right Parieto-occipital pontine, right Superior Longitudinal fascicle II, and left Superior Longitudinal Fascicle III had a main effect on recovery groups. However, none of these main effects survived FDR correction (P > 0.050).

## SUPPLEMENTAL TABLES

**Supplemental Table 1. Demographic characteristics of included and excluded concussed participants.**

| ***Demographic Characteristics*** | ***Total Concussed sample (N=50)*** | ***Included (N=42)*** | ***Excluded (N=8)*** | ***t(48)* or *χ2*** | ***P value^1^*** |
| --- | --- | --- | --- | --- | --- |
| Age, mean [SD], y | 15.6 [1.6] | 15.5 [1.7] | 15.9 [0.9] | 0.8 | 0.426 |
| Sex |  |  |  |  |  |
| Male, No. (%) | 28 (56.0%) | 24 (57.1%) | 4 (50.0%) | 0.1 | 0.709 |
| Female, No. (%) | 22 (44.0%) | 18 (42.9%) | 4 (50.0%) |  |  |
| Race |  |  |  |  |  |
| Caucasian, No. (%) | 44 (88.0%) | 37 (88.1%) | 7 (87.5%) | < 0.1 | 0.962 |
| Non-Caucasian, No. (%) | 6 (12.0%) | 5 (11.9%) | 1 (12.5%) |  |  |

^1^ P values ⩽ 0.050 are reported in bold characters and P values with a trend towards statistical significance are reported in italics

**Supplemental Table 2. Demographic and clinical characteristics of concussed and healthy participants.**

| ***Demographic Characteristics*** | ***Total sample (N=84)*** | ***HC (N=42)*** | ***Concussed (N=42)*** | ***t(82)* or *χ2*** | ***P value^1^*** |
| --- | --- | --- | --- | --- | --- |
| Age, mean [SD], y | 15.3 [1.7] | 15.1 [1.6] | 15.5 [1.7] | -1.1 | 0.291 |
| Sex |  |  |  |  |  |
| Male, No. (%) | 47 (55.9%) | 23 (54.8%) | 24 (57.1%) | 0.1 | 0.826 |
| Female, No. (%) | 37 (44.1%) | 19 (45.2%) | 18 (42.9%) |  |  |
| Race |  |  |  |  |  |
| Caucasian, No. (%) | 67 (79.8%) | 30 (71.4%) | 37 (88.1%) | 3.6 | *0.057* |
| Non-Caucasian, No. (%) | 17 (20.2%) | 12 (28.6%) | 5 (11.9%) |  |  |
| ***Clinical Characteristics*** | ***Total sample (N=84)*** | ***HC (N=42)*** | ***Concussed (N=42)*** | ***t(82) or t(77)***^2^ | ***P value^1^*** |
| BIS total score, mean [SD] | 56.6 [8.5] | 54.5 [9.2] | 58.7 [7.3] | -2.3 | **0.022** |
| SCARED total score, mean [SD] | 10.1 [10.0] | 8.4 [8.2] | 11.9 [11.2] | -1.7 | 0.103 |
| CALS, total score, mean [SD] | 4.5 [6.5] | 3.3 [5.0] | 5.7 [7.5] | -1.7 | *0.091* |
| CDRS^2^ total score, mean [SD] | 20.6 [4.4] | 19.8 [4.0] | 21.2 [4.7] | -1.4 | 0.174 |

Abbreviations: HC, Healthy Controls; BIS, Barratt Impulsivity Scale; SCARED, Screen for Child Anxiety Related Disorders; CALS, Children’s Affective Lability Scale; CDRS, Children's Depression Rating Scale.

^1^ P values ⩽ 0.050 are reported in bold characters and P values with a trend towards statistical significance are reported in italics

^2^ Five HC had missing CDRS data.

**Supplemental Table 3. Clinical characteristics of concussed participants.**

| ***Clinical Characteristics*** | ***Total sample (N=42)*** | ***SHORT (N=21)*** | ***LONG (N=21)*** | ***t(40)*** | ***P value^1^*** |
| --- | --- | --- | --- | --- | --- |
| BIS total score, mean [SD] | 58.7 [7.3] | 58.4 [7.3] | 59.0 [7.4] | -0.2 | 0.820 |
| SCARED total score, mean [SD] | 11.9 [11.2] | 11.2 [11.5] | 12.6 [11.2] | -0.4 | 0.685 |
| CALS, total score, mean [SD] | 5.7 [7.5] | 6.1 [8.1] | 5.3 [7.1] | 0.3 | 0.746 |
| CDRS total score, mean [SD] | 21.2 [4.7] | 20.9 [4.5] | 21.5 [5.0] | -0.4 | 0.699 |

Abbreviations: HC, Healthy Controls; BIS, Barratt Impulsivity Scale; SCARED, Screen for Child Anxiety Related Disorders; CALS, Children’s Affective Lability Scale; CDRS, Children's Depression Rating Scale; SHORT, Short Recovery; LONG, Long Recovery.

^1^ P values ⩽ 0.050 are reported in bold characters and P values with a trend towards statistical significance are reported in italics

**Supplemental Table 4. Statistical results for main hypothesis analyses.**

| *Logistic regression models^1^* | | | | | |
| --- | --- | --- | --- | --- | --- |
| ***Variable*** | ***OR*** | ***95% CI*** | | ***P-value^2^*** | ***FDR P-value^2,3^*** |
| Left ILF - mean FA | 0.36 | 0.15 | 0.91 | **0.030** | **0.030** |
| Right ILF - mean FA | 0.28 | 0.10 | 0.83 | **0.021** | **0.025** |
| Left IFOF - mean FA | 0.21 | 0.07 | 0.66 | **0.008** | **0.022** |
| Right IFOF - mean FA | 0.30 | 0.11 | 0.83 | **0.020** | **0.025** |
| Left UF - mean FA | 0.26 | 0.09 | 0.74 | **0.011** | **0.022** |
| Right UF - mean FA | 0.28 | 0.10 | 0.73 | **0.010** | **0.022** |
| *Focal abnormalities in white matter tracts of interest* | | | | | |
| ***Variable*** | ***OR*** | ***95% CI*** | | ***P-value^2^*** | ***FDR P-value^2,3^*** |
| Left ILF - Temporal cluster FA | 0.13 | 0.03 | 0.54 | **0.005** | **0.011** |
| Right ILF - Temporal cluster FA | 0.16 | 0.04 | 0.63 | **0.009** | **0.011** |
| Left IFOF - Frontal cluster FA | 0.30 | 0.11 | 0.80 | **0.016** | **0.016** |
| Left IFOF - Middle cluster FA | 0.14 | 0.03 | 0.55 | **0.005** | **0.011** |
| Right IFOF - Middle cluster FA | 0.22 | 0.07 | 0.67 | **0.008** | **0.011** |
| Left UF - Temporal cluster FA | 0.25 | 0.09 | 0.66 | **0.005** | **0.011** |
| Right UF - Temporal cluster FA | 0.28 | 0.11 | 0.70 | **0.006** | **0.011** |

Abbreviations: ILF, Inferior Longitudinal Fasciculus; IFOF, Inferior Fronto-Occipital Fasciculus; UF, Uncinate Fasciculus; FA; Fractional Anisotropy; OR, Odds Ratio; CI, Confidence Interval; FDR, False Discovery Rate.

^1^ Additional analysis involving other white matter tracts are reported on Supplemental Table 10.

^2^ P values ⩽ 0.050 are reported in bold characters and P values with a trend towards statistical significance are reported in italics.

^3^ FDR corrected P values.

**Supplemental Table 5. Radial and Axial diffusivity – Statistical test results.**

|  | *Axial Diffusivity (AD)* | | | | | *Radial Diffusivity (RD)* | | | | |
| --- | --- | --- | --- | --- | --- | --- | --- | --- | --- | --- |
| ***Variable*** | ***OR*** | ***95% CI*** | | ***P-value*^1^** | ***FDR P-value^1,2^*** | ***OR*** | ***95% CI*** | | ***P-value*^1^** | ***FDR P-value^1,2^*** |
| Left ILF - Temporal cluster | 1.13 | 0.59 | 2.14 | 0.720 | 0.999 | 6.95 | 1.55 | 31.20 | **0.011** | **0.026** |
| Right ILF - Temporal cluster | 1.12 | 0.60 | 2.10 | 0.728 | 0.999 | 8.68 | 1.82 | 41.33 | **0.007** | **0.025** |
| Left IFOF - Frontal cluster | 1.23 | 0.70 | 2.17 | 0.475 | 0.999 | 3.84 | 1.07 | 13.77 | **0.039** | **0.039** |
| Left IFOF - Middle cluster | 1.02 | 0.54 | 1.94 | 0.947 | 0.999 | 10.14 | 2.11 | 48.71 | **0.004** | **0.025** |
| Right IFOF - Middle cluster | 1.00 | 0.54 | 1.85 | 0.999 | 0.999 | 4.02 | 1.22 | 13.23 | **0.022** | **0.031** |
| Left UF - Temporal cluster | 0.99 | 0.55 | 1.78 | 0.974 | 0.999 | 2.73 | 1.06 | 7.01 | **0.038** | **0.039** |
| Right UF - Temporal cluster | 1.24 | 0.65 | 2.38 | 0.513 | 0.999 | 3.24 | 1.23 | 8.59 | **0.018** | **0.031** |

Abbreviations: ILF, Inferior Longitudinal Fasciculus; IFOF, Inferior Fronto-Occipital Fasciculus; UF, Uncinate Fasciculus; FDR, False Discovery Rate.

^1^ P values ⩽ 0.050 are reported in bold characters.

^2^ FDR corrected P values.

**Supplemental Table 6. Statistical results for between-group analyses.**

| *Between-group analyses^1^ – LONG vs HC* | | | |
| --- | --- | --- | --- |
| **Variable** | ***F[1,59]*** | ***P-value*^2^** | ***FDR P-value^2,3^*** |
| Left ILF - Temporal cluster FA | 9.7 | **0.003** | **0.011** |
| Right ILF - Temporal cluster FA | 13.6 | **<0.001** | **<0.001** |
| Left IFOF - Frontal cluster FA | 1.0 | 0.327 | 0.412 |
| Left IFOF - Middle cluster FA | 6.4 | **0.014** | **0.025** |
| Right IFOF - Middle cluster FA | 6.8 | **0.012** | **0.025** |
| Left UF - Temporal cluster FA | <0.1 | 0.874 | 0.874 |
| Right UF - Temporal cluster FA | 0.9 | 0.353 | 0.412 |
| *Between-group analyses - SHORT vs HC* | | | |
| **Variable** | ***F[1,59]*** | ***P-value*^2^** | ***FDR P-value^2,3^*** |
| Left ILF - Temporal cluster FA | 0.2 | 0.651 | 0.689 |
| Right ILF - Temporal cluster FA | 0.9 | 0.354 | 0.557 |
| Left IFOF - Frontal cluster FA | 1.6 | 0.214 | 0.499 |
| Left IFOF - Middle cluster FA | 0.2 | 0.689 | 0.689 |
| Right IFOF - Middle cluster FA | 0.7 | 0.398 | 0.557 |
| Left UF - Temporal cluster FA | 7.4 | **0.009** | *0.063* |
| Right UF - Temporal cluster FA | 4.1 | **0.047** | 0.165 |

Abbreviations: ILF, Inferior Longitudinal Fasciculus; IFOF, Inferior Fronto-Occipital Fasciculus; UF, Uncinate Fasciculus; HC, Healthy Controls; SHORT, Short Recovery; LONG, Prolonged Recovery; ILF, Inferior Longitudinal Fasciculus; UF, Uncinate Fasciculus; FDR, False Discovery Rate.

^1^ Additional between-group analyses compared Concussed participants (N=42; SHORT and LONG recovery groups combined) and HC and revealed no between-group differences (P>0.050).

^2^ P values ⩽ 0.050 are reported in bold characters and P values with a trend towards statistical significance are reported in italics.

^3^ FDR corrected P values.

**Supplemental Table 7. Main effect of FA of focal abnormalities on recovery time (range=9-150 days).**

| **Variable** | **B** | ***P-value*^1^** | ***FDR P-value^1,2^*** |
| --- | --- | --- | --- |
| Left ILF - Temporal cluster FA | -17.57 | **0.006** | **0.016** |
| Right ILF - Temporal cluster FA | -16.43 | **0.001** | **0.007** |
| Left IFOF - Frontal cluster FA | -13.37 | **0.024** | **0.028** |
| Left IFOF - Middle cluster FA | -14.72 | **0.040** | **0.040** |
| Right IFOF - Middle cluster FA | -14.13 | **0.021** | **0.028** |
| Left UF - Temporal cluster FA | -14.95 | **0.014** | **0.025** |
| Right UF - Temporal cluster FA | -15.44 | **0.007** | **0.016** |

Abbreviations: ILF, Inferior Longitudinal Fasciculus; IFOF, Inferior Fronto-Occipital Fasciculus; UF, Uncinate Fasciculus; FA, Fractional Anisotropy; FDR, False Discovery Rate.

^1^ P values ⩽ 0.050 are reported in bold characters and P values with a trend towards statistical significance are reported in italics

^2^ FDR corrected P values.

**Supplemental Table 8. Relationships between FA of node clusters and clinical variables in the concussed group.**

**Supplemental Table 8A. ILF relationships.**

|  | Left ILF - Temporal cluster FA | | | Right ILF - Temporal cluster FA | | |
| --- | --- | --- | --- | --- | --- | --- |
|  |  |  |  |  |  |  |
| **Variables** | ***r or rho*** | ***P-value*^1^** | ***FDR P-value^1,2^*** | ***r or rho*** | ***P-value*^1^** | ***FDR P-value^1,2^*** |
| Composite scores |  |  |  |  |  |  |
| Verbal memory | 0.13 | 0.402 | 0.653 | 0.09 | 0.564 | 0.832 |
| Visual memory | 0.21 | 0.172 | 0.559 | 0.18 | 0.264 | 0.832 |
| Visual motor speed | 0.12 | 0.455 | 0.657 | 0.02 | 0.886 | 0.904 |
| Reaction time | -0.14 | 0.363 | 0.653 | 0.15 | 0.329 | 0.832 |
| Symptom factors |  |  |  |  |  |  |
| Affective factor | 0.04 | 0.816 | 0.963 | -0.15 | 0.341 | 0.832 |
| Somatic factor | 0.00 | 0.981 | 0.981 | -0.08 | 0.594 | 0.832 |
| Sleep factor | -0.16 | 0.313 | 0.653 | -0.11 | 0.486 | 0.832 |
| Cognitive-migraine-fatigue factor | -0.02 | 0.889 | 0.963 | -0.15 | 0.354 | 0.832 |
| VOMS total symptom score | -0.24 | 0.122 | 0.529 | -0.07 | 0.676 | 0.832 |
| BIS total score | -0.05 | 0.771 | 0.963 | 0.06 | 0.687 | 0.832 |
| SCARED total score | 0.31 | **0.046** | 0.529 | -0.18 | 0.250 | 0.832 |
| CALS, total score | 0.25 | 0.109 | 0.529 | -0.02 | 0.904 | 0.904 |
| CDRS total score | 0.18 | 0.248 | 0.645 | -0.06 | 0.704 | 0.832 |

**Supplemental Table 8B. IFOF relationships.**

|  | Left IFOF - Frontal cluster FA | | | Left IFOF - Middle cluster FA | | | Right IFOF - Middle cluster FA | | |
| --- | --- | --- | --- | --- | --- | --- | --- | --- | --- |
|  |  |  |  |  |  |  |  |  |  |
| **Variables** | ***r or rho*** | ***P-value*^1^** | ***FDR P-value^1,2^*** | ***r or rho*** | ***P-value*^1^** | ***FDR P-value^1,2^*** | ***r or rho*** | ***P-value*^1^** | ***FDR P-value^1,2^*** |
| Composite scores |  |  |  |  |  |  |  |  |  |
| Verbal memory | 0.29 | *0.059* | 0.449 | 0.14 | 0.373 | 0.560 | 0.04 | 0.801 | 0.910 |
| Visual memory | 0.28 | *0.069* | 0.449 | 0.01 | 0.949 | 0.949 | 0.09 | 0.583 | 0.910 |
| Visual motor speed | 0.02 | 0.884 | 0.884 | 0.10 | 0.537 | 0.635 | -0.07 | 0.655 | 0.910 |
| Reaction time | 0.08 | 0.615 | 0.841 | -0.14 | 0.388 | 0.560 | -0.02 | 0.913 | 0.910 |
| Symptom factors |  |  |  |  |  |  |  |  |  |
| Affective factor | 0.10 | 0.519 | 0.841 | 0.12 | 0.456 | 0.593 | 0.08 | 0.605 | 0.910 |
| Somatic factor | 0.06 | 0.712 | 0.841 | 0.20 | 0.196 | 0.560 | 0.09 | 0.567 | 0.910 |
| Sleep factor | -0.08 | 0.615 | 0.841 | -0.18 | 0.262 | 0.560 | -0.11 | 0.493 | 0.910 |
| Cognitive-migraine-fatigue factor | 0.10 | 0.524 | 0.841 | -0.02 | 0.905 | 0.949 | -0.03 | 0.844 | 0.910 |
| VOMS total symptom score | 0.06 | 0.704 | 0.841 | 0.14 | 0.367 | 0.560 | -0.07 | 0.669 | 0.910 |
| BIS total score | -0.04 | 0.796 | 0.862 | -0.16 | 0.327 | 0.560 | -0.15 | 0.353 | 0.910 |
| SCARED total score | 0.17 | 0.282 | 0.841 | 0.26 | *0.099* | 0.524 | 0.15 | 0.352 | 0.910 |
| CALS, total score | 0.08 | 0.595 | 0.841 | 0.28 | *0.072* | 0.524 | 0.08 | 0.613 | 0.910 |
| CDRS total score | 0.11 | 0.476 | 0.841 | 0.24 | 0.121 | 0.524 | 0.04 | 0.817 | 0.910 |

**Supplemental Table 8C. UF relationships.**

|  | Left UF - Temporal cluster FA | | | Right UF - Temporal cluster FA | | |
| --- | --- | --- | --- | --- | --- | --- |
|  |  |  |  |  |  |  |
| **Variables** | ***r or rho*** | ***P-value*^1^** | ***FDR P-value^1,2^*** | ***r or rho*** | ***P-value*^1^** | ***FDR P-value^1,2^*** |
| Composite scores |  |  |  |  |  |  |
| Verbal memory | 0.14 | 0.367 | 0.741 | 0.36 | **0.018** | 0.117 |
| Visual memory | 0.06 | 0.720 | 0.780 | 0.37 | **0.018** | 0.117 |
| Visual motor speed | 0.00 | 0.996 | 0.996 | 0.23 | 0.141 | 0.367 |
| Reaction time | 0.08 | 0.637 | 0.780 | -0.19 | 0.236 | 0.485 |
| Symptom factors |  |  |  |  |  |  |
| Affective factor | 0.18 | 0.259 | 0.710 | -0.18 | 0.261 | 0.485 |
| Somatic factor | -0.07 | 0.673 | 0.780 | -0.08 | 0.632 | 0.747 |
| Sleep factor | -0.19 | 0.223 | 0.710 | 0.01 | 0.957 | 0.971 |
| Cognitive-migraine-fatigue factor | -0.17 | 0.273 | 0.710 | -0.24 | 0.123 | 0.367 |
| VOMS total symptom score | -0.20 | 0.206 | 0.710 | -0.33 | **0.032** | 0.139 |
| BIS total score | -0.19 | 0.219 | 0.710 | 0.01 | 0.971 | 0.971 |
| SCARED total score | 0.12 | 0.456 | 0.741 | 0.16 | 0.309 | 0.502 |
| CALS, total score | 0.12 | 0.450 | 0.741 | 0.09 | 0.563 | 0.732 |
| CDRS total score | 0.06 | 0.706 | 0.780 | 0.15 | 0.352 | 0.508 |

Abbreviations: FA, Fractional Anisotropy; ILF, Inferior Longitudinal Fasciculus; IFOF, Inferior Fronto-Occipital Fasciculus; UF, Uncinate Fasciculus; VOMS, Vestibular/Ocular-Motor Screening; SCARED, Screen for Child Anxiety Related Disorders; CALS, Children’s Affective Lability Scale; CDRS, Children's Depression Rating Scale; FDR, False Discovery Rate;

^1^ P values ⩽ 0.050 are reported in bold characters and P values with a trend towards statistical significance are reported in italics

^2^ FDR corrected P values.

**Supplemental Table 9. Kolmogorov-Smirnov tests of FA distribution between recovery groups.**

| Node cluster | D | P^1^ |
| --- | --- | --- |
| Left ILF - Temporal cluster FA | 0.3 | 0.194 |
| Right ILF - Temporal cluster FA | 0.5 | **0.017** |
| Left IFOF - Frontal cluster FA | 0.3 | 0.358 |
| Left IFOF - Middle cluster FA | 0.3 | 0.194 |
| Right IFOF - Middle cluster FA | 0.3 | 0.194 |
| Left UF - Temporal cluster FA | 0.3 | 0.358 |
| Right UF - Temporal cluster FA | 0.4 | *0.095* |

Abbreviations: FA, Fractional Anisotropy; ILF, Inferior Longitudinal Fasciculus; IFOF, Inferior Fronto-Occipital Fasciculus; UF, Uncinate Fasciculus.

^1^ P values ⩽ 0.050 are reported in bold characters and P values with a trend towards statistical significance are reported in italics.

**Supplemental Table 10. Effects of dMRI movements.**

|  | Averaged translation | | | Averaged rotation | | |
| --- | --- | --- | --- | --- | --- | --- |
|  |  |  |  |  |  |  |
| **dMRI movements** | ***rho*** | ***P-value*^1^** | ***FDR P-value^1,2^*** | ***rho*** | ***P-value*^1^** | ***FDR P-value^1,2^*** |
| Left ILF - Temporal cluster FA | -0.13 | 0.422 | 0.646 | -0.25 | 0.116 | 0.256 |
| Right ILF - Temporal cluster FA | 0.01 | 0.961 | 0.961 | -0.11 | 0.483 | 0.483 |
| Left IFOF - Frontal cluster FA | 0.11 | 0.495 | 0.646 | -0.23 | 0.146 | 0.256 |
| Left IFOF - Middle cluster FA | -0.23 | 0.138 | 0.646 | -0.28 | **0.017** | 0.119 |
| Right IFOF - Middle cluster FA | -0.19 | 0.236 | 0.646 | -0.18 | 0.125 | 0.256 |
| Left UF - Temporal cluster FA | -0.13 | 0.413 | 0.646 | -0.19 | 0.233 | 0.326 |
| Right UF - Temporal cluster FA | -0.09 | 0.554 | 0.646 | -0.14 | 0.360 | 0.420 |

Abbreviations: ILF, Inferior Longitudinal Fasciculus; IFOF, Inferior Fronto-Occipital Fasciculus; UF, Uncinate Fasciculus; FDR, False Discovery Rate.

^1^ P values ⩽ 0.050 are reported in bold characters.

^2^ FDR corrected P values.

**Supplemental Table 11. Exploratory analyses in other major white matter tracts.**

| **Variable** | ***OR*** | ***95% CI*** | | ***P-value*^1^** | ***FDR P-value^1,2^*** |
| --- | --- | --- | --- | --- | --- |
| Left Anterior thalamic radiation - mean FA | 0.62 | 0.30 | 1.28 | 0.198 | 0.234 |
| Right Anterior thalamic radiation - mean FA | 0.41 | 0.17 | 1.02 | *0.055* | 0.142 |
| Left Cingulum Bundle – mean FA | 0.57 | 0.27 | 1.18 | 0.128 | 0.192 |
| Right Cingulum Bundle – mean FA | 0.50 | 0.24 | 1.08 | *0.077* | 0.158 |
| Corpus Callosum - Rostrum - mean FA | 0.81 | 0.42 | 1.57 | 0.538 | 0.552 |
| Corpus Callosum - Genu - mean FA | 1.02 | 0.79 | 1.22 | *0.097* | 0.164 |
| Corpus Callosum - Rostral body - mean FA | 0.64 | 0.32 | 1.26 | 0.194 | 0.234 |
| Corpus Callosum - Anterior midbody - mean FA | 0.38 | 0.15 | 0.96 | **0.041** | 0.142 |
| Corpus Callosum - Posterior midbody - mean FA | 0.44 | 0.19 | 0.99 | **0.047** | 0.142 |
| Corpus Callosum - Isthmus - mean FA | 0.52 | 0.23 | 1.16 | 0.109 | 0.170 |
| Corpus Callosum - Splenium - mean FA | 0.45 | 0.21 | 0.93 | **0.032** | 0.142 |
| Left Corticospinal tract - mean FA | 0.74 | 0.38 | 1.43 | 0.367 | 0.387 |
| Right Corticospinal tract - mean FA | 0.59 | 0.29 | 1.19 | 0.138 | 0.196 |
| Left Fronto-pontine tract - mean FA | 0.72 | 0.37 | 1.41 | 0.334 | 0.362 |
| Right Fronto-pontine tract - mean FA | 0.59 | 0.29 | 1.23 | 0.159 | 0.207 |
| Left Optic radiation - mean FA | 0.45 | 0.20 | 1.01 | *0.053* | 0.142 |
| Right Optic radiation - mean FA | 0.31 | 0.12 | 0.81 | **0.016** | 0.142 |
| Left Parieto‐occipital pontine - mean FA | 0.64 | 0.32 | 1.29 | 0.210 | 0.241 |
| Right Parieto‐occipital pontine - mean FA | 0.36 | 0.14 | 0.92 | **0.032** | 0.142 |
| Left Superior longitudinal fascicle I - mean FA | 0.48 | 0.23 | 1.04 | *0.062* | 0.142 |
| Right Superior longitudinal fascicle I - mean FA | 0.43 | 0.18 | 1.03 | *0.058* | 0.142 |
| Left Superior longitudinal fascicle II - mean FA | 0.53 | 0.25 | 1.12 | *0.096* | 0.164 |
| Right Superior longitudinal fascicle II - mean FA | 0.37 | 0.16 | 0.89 | **0.026** | 0.142 |
| Left Superior longitudinal fascicle III - mean FA | 0.40 | 0.17 | 0.94 | **0.036** | 0.142 |
| Right Superior longitudinal fascicle III - mean FA | 0.51 | 0.23 | 1.11 | *0.089* | 0.164 |
| Left Arcuate fascicle - mean FA | 0.60 | 0.30 | 1.20 | 0.146 | 0.196 |
| Right Arcuate fascicle - mean FA | 0.37 | 0.15 | 0.90 | **0.029** | 0.142 |
| Left Superior thalamic radiation - mean FA | 0.50 | 0.23 | 1.06 | *0.071* | 0.154 |
| Right Superior thalamic radiation - mean FA | 0.48 | 0.22 | 1.04 | *0.061* | 0.142 |
| Left Thalamo-premotor - mean FA | 0.54 | 0.26 | 1.14 | 0.106 | 0.170 |
| Right Thalamo-premotor - mean FA | 0.56 | 0.26 | 1.22 | 0.144 | 0.196 |
| Left Thalamo-parietal - mean FA | 0.43 | 0.19 | 0.97 | **0.041** | 0.142 |
| Right Thalamo-parietal - mean FA | 0.29 | 0.10 | 0.84 | **0.022** | 0.142 |
| Left Thalamic-occipital - mean FA | 0.45 | 0.20 | 1.00 | *0.051* | 0.142 |
| Right Thalamic-occipital - mean FA | 0.34 | 0.13 | 0.85 | **0.021** | 0.142 |
| Left Striato-fronto-orbital - mean FA | 0.90 | 0.46 | 1.73 | 0.745 | 0.745 |
| Right Striato-fronto-orbital - mean FA | 0.66 | 0.33 | 1.31 | 0.232 | 0.259 |
| Left Striato-premotor - mean FA | 0.52 | 0.24 | 1.12 | *0.094* | 0.164 |
| Right Striato-premotor - mean FA | 0.61 | 0.29 | 1.27 | 0.187 | 0.234 |

Abbreviations: FA; Fractional Anisotropy; OR, Odds Ratio; CI, Confidence Interval; FDR, False Discovery Rate;

^1^ P values ⩽ 0.050 are reported in bold characters and P values with a trend towards statistical significance are reported in italics

^2^ FDR corrected P values.

## REFERENCES

1 Sheehan DV, Sheehan KH, Shytle RD, Janavs J, Bannon Y, Rogers JE, et al. Reliability and validity of the mini international neuropsychiatric interview for children and adolescents (MINI-KID). The Journal of clinical psychiatry. 2010.

2 Kaufman J, Birmaher B, Brent D, Rao U, Flynn C, Moreci P, et al. Schedule for affective disorders and schizophrenia for school-age children-present and lifetime version (K-SADS-PL): initial reliability and validity data. Journal of the American Academy of Child & Adolescent Psychiatry. 1997;36(7):980-88.

3 Patton JH, Stanford MS, Barratt ES. Factor structure of the Barratt impulsiveness scale. Journal of clinical psychology. 1995;51(6):768-74.

4 Birmaher B, Brent DA, Chiappetta L, Bridge J, Monga S, Baugher M. Psychometric properties of the Screen for Child Anxiety Related Emotional Disorders (SCARED): a replication study. Journal of the American academy of child & adolescent psychiatry. 1999;38(10):1230-36.

5 Gerson AC, Gerring JP, Freund L, Joshi PT, Capozzoli J, Brady K, et al. The Children's Affective Lability Scale: a psychometric evaluation of reliability. Psychiatry research. 1996;65(3):189-98.

6 Poznanski EO, Mokros HB. Children's depression rating scale, revised (CDRS-R)*.* Western Psychological Services Los Angeles; 1996.

7 Andersson JL, Skare S, Ashburner J. How to correct susceptibility distortions in spin-echo echo-planar images: application to diffusion tensor imaging. Neuroimage. 2003;20(2):870-88.

8 Smith SM, Jenkinson M, Woolrich MW, Beckmann CF, Behrens TE, Johansen-Berg H, et al. Advances in functional and structural MR image analysis and implementation as FSL. Neuroimage. 2004;23:S208-S19.

9 Yendiki A, Koldewyn K, Kakunoori S, Kanwisher N, Fischl B. Spurious group differences due to head motion in a diffusion MRI study. Neuroimage. 2014;88:79-90.
